# Supplementary material for: The quaternary architecture of RARβ–RXRα heterodimer facilitates domain–domain signal transmission
Source: Nat Commun. 2017 Oct 11;8:868. doi: 10.1038/s41467-017-00981-y (PMC5636793; doi:10.1038/s41467-017-00981-y)
Supplement: Supplementary file 1 — Supplementary Information [file 41467_2017_981_MOESM1_ESM.pdf]

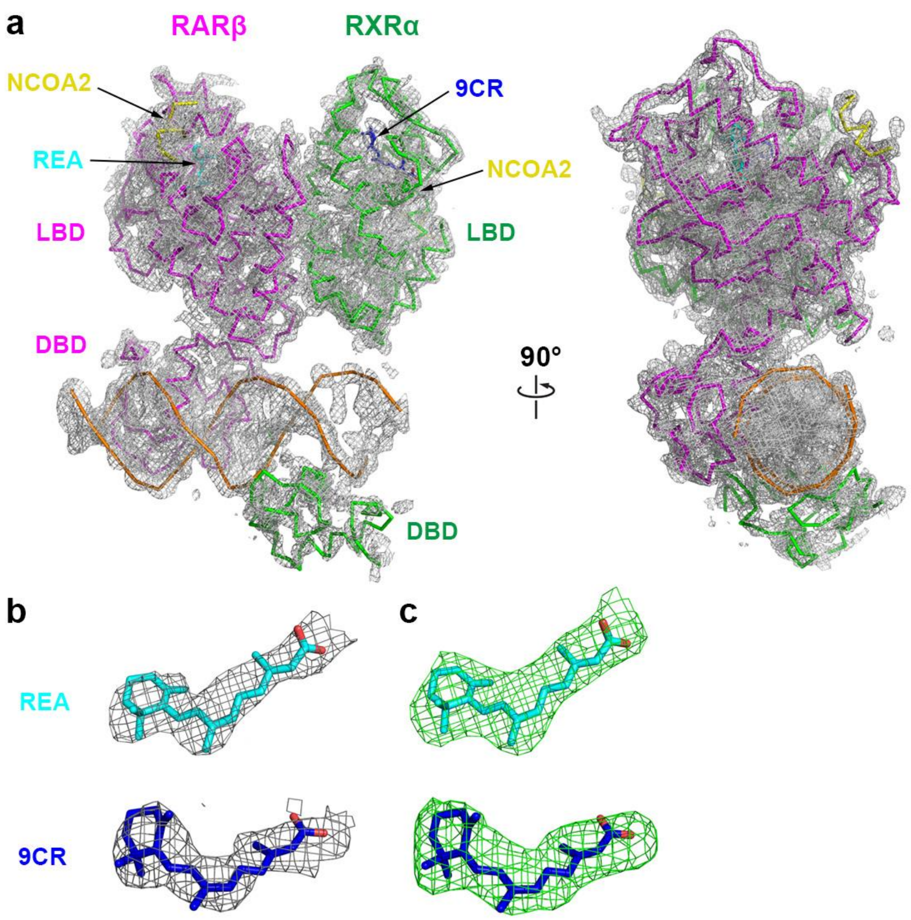

Supplementary Figure 1 Electron density maps of the RARβ-RXRα-DNA complex. (**a**) $2F_O - F_C$ map contoured at 1.0σ covers the overall RARβ-RXRα-DNA complex in two views. The colors used for RARβ, RXRα, DNA, NCOA2 peptides, REA, 9CR and density are magenta, green, orange, yellow, cyan, blue and gray, respectively. (**b** and **c**) The $2F_O - F_C$ maps at 1.0σ (**b**) and polder OMIT maps at 3.0σ (**c**) of two ligands REA and 9CR are shown in gray and green colors, respectively.

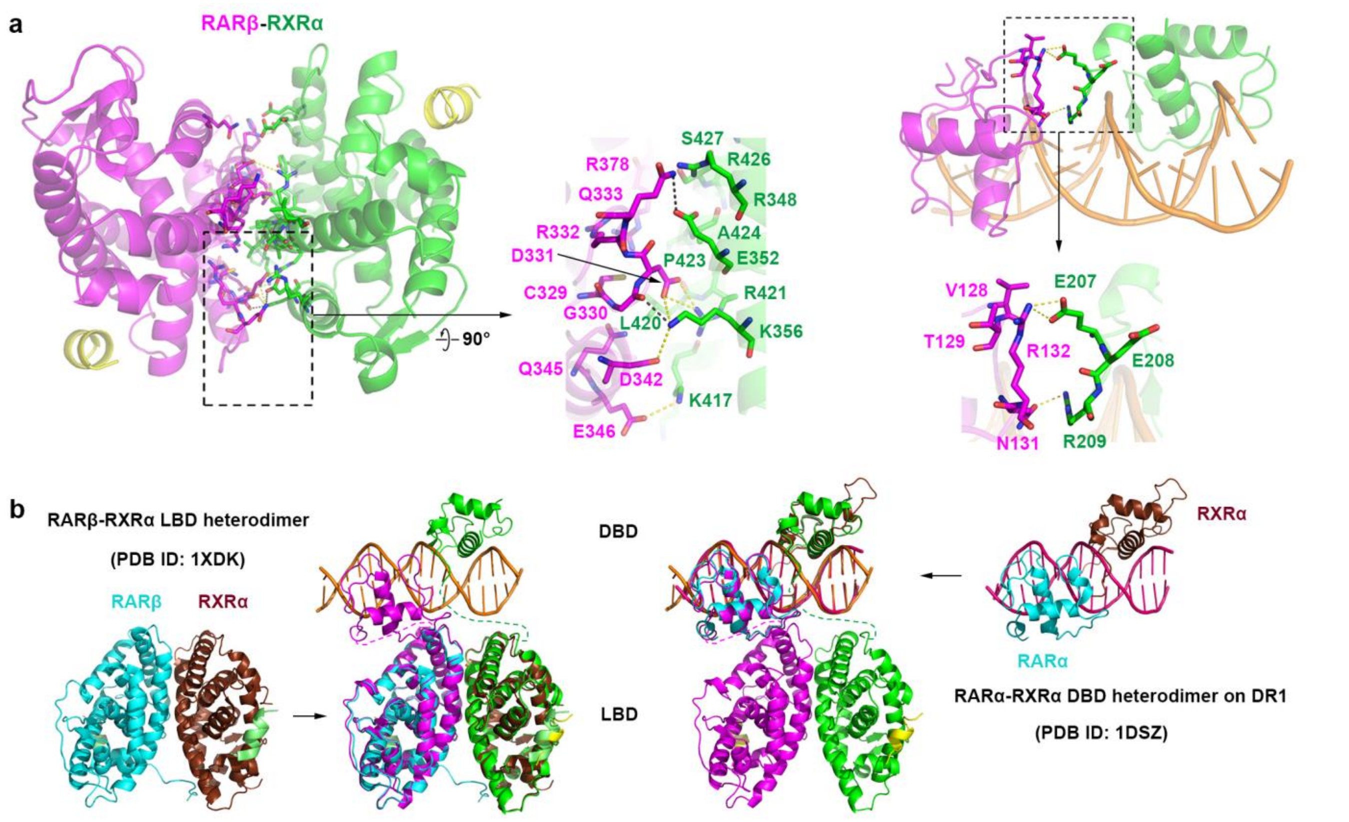

Supplementary Figure 2 Dimerization of the multi-domain RARβ-RXRα complex. (**a**) The dimerization interfaces between two LBDs (left) and two DBDs (right), with enlarged views showing representative details of the interactions. These interfaces are mainly mediated by salt bridges (yellow dotted lines), hydrogen bonds (gray dotted lines) and hydrophobic interactions. (**b**) Comparison between multi-domain RARβ-RXRα complex and isolated DBD or LBD heterodimers. Structure superimpositions of RARβ-RXRα-DNA complex with previously published RARβ-RXRα LBD heterodimer (left) and RARα-RXRα DBD heterodimer on DR1 DNA (right).

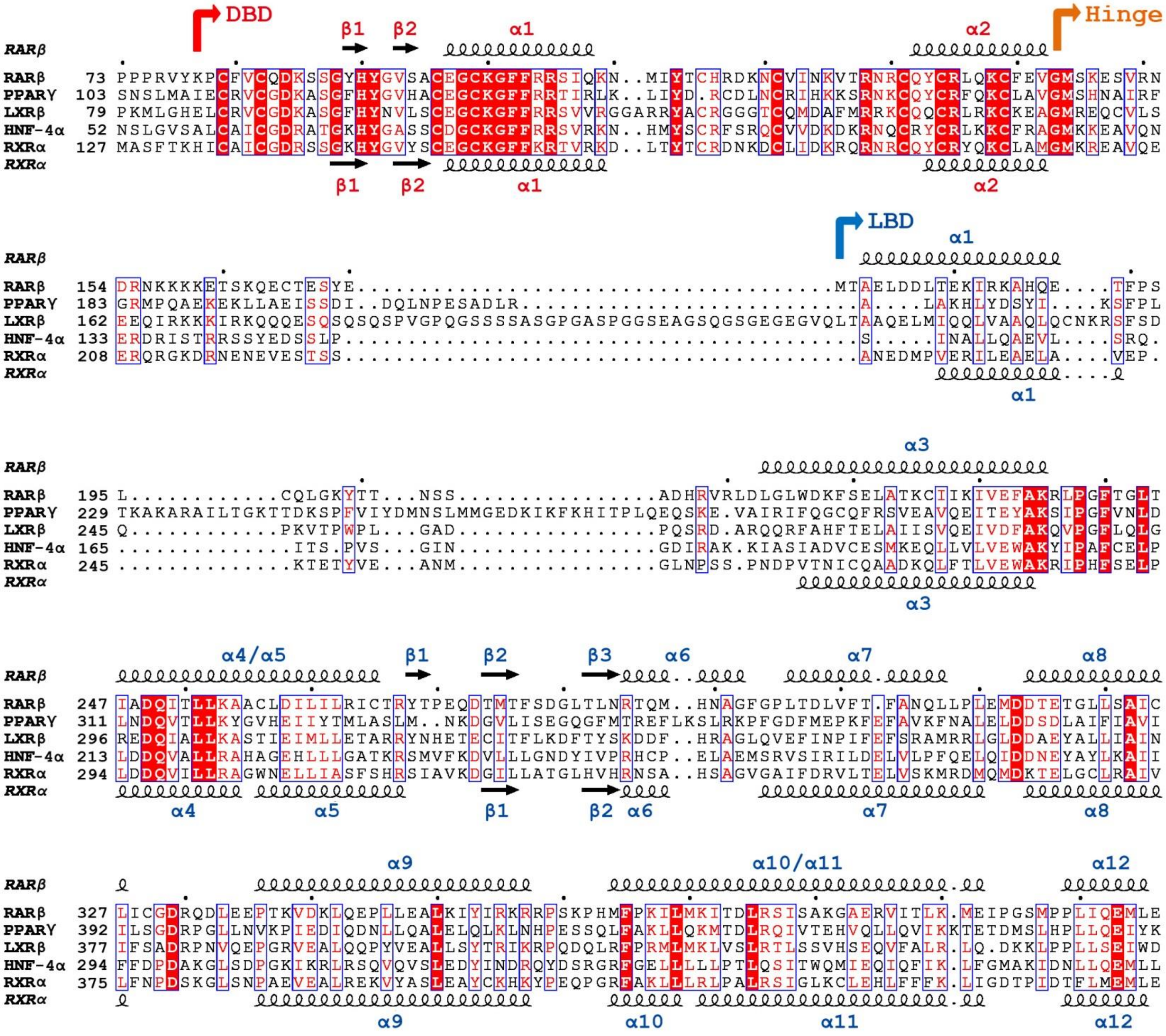

Supplementary Figure 3 Structure-based sequence alignment of human RARβ, PPARγ, LXRβ, HNF-4α and RXRα proteins. The starting positions of DBD, hinge and LBD segments are labelled in red, orange and blue, respectively. The secondary structure components of RARβ are shown above the alignment, while those of RXRα shown below.

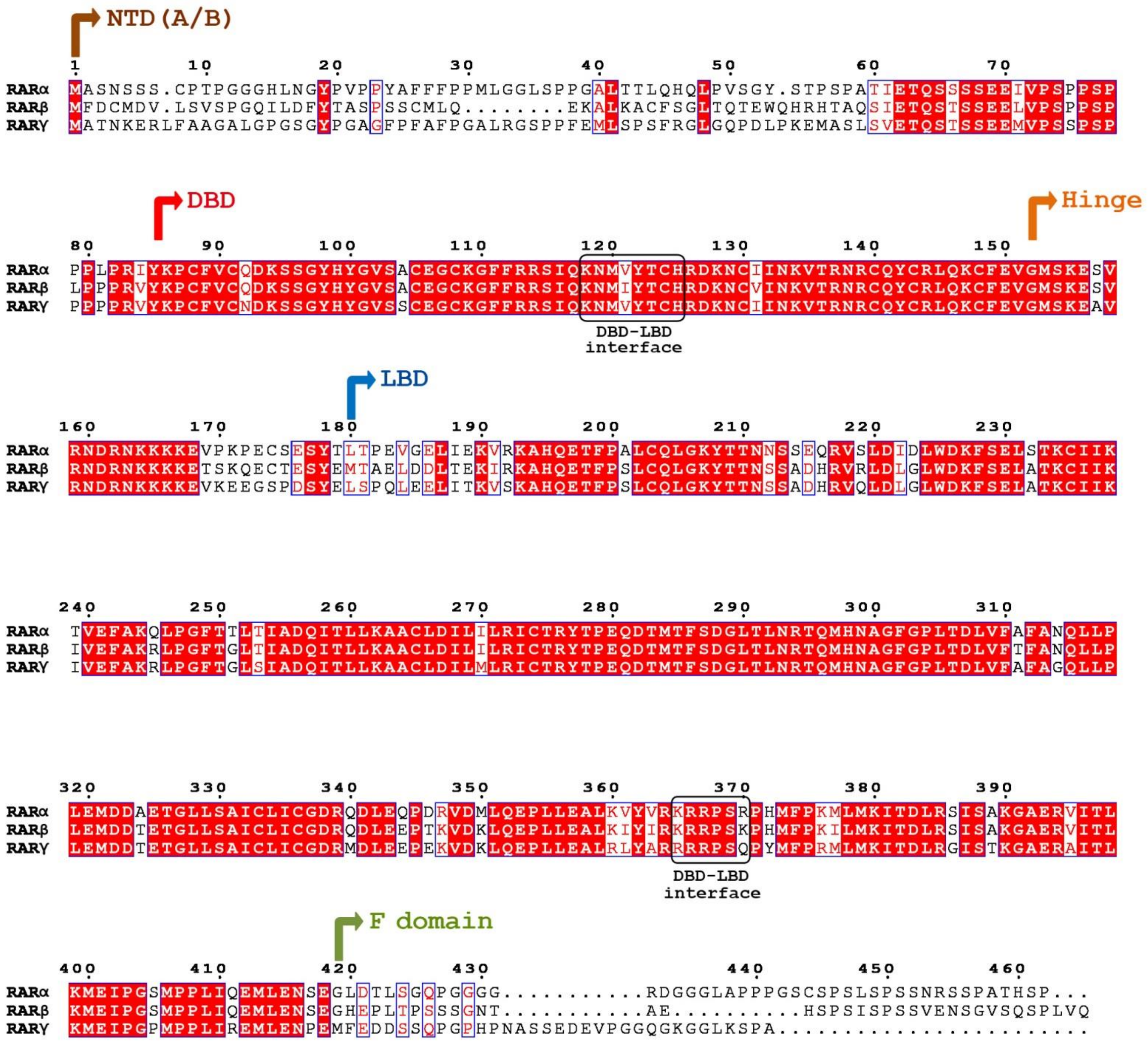

Supplementary Figure 4 Sequence alignment of human full-length RARα, RARβ and RARγ proteins. The starting positions of NTD (A/B domain), DBD, hinge, LBD and F domain segments are labelled in brown, red, orange, blue and green, respectively. The regions involved in DBD-LBD interface are also indicated by black boxes.

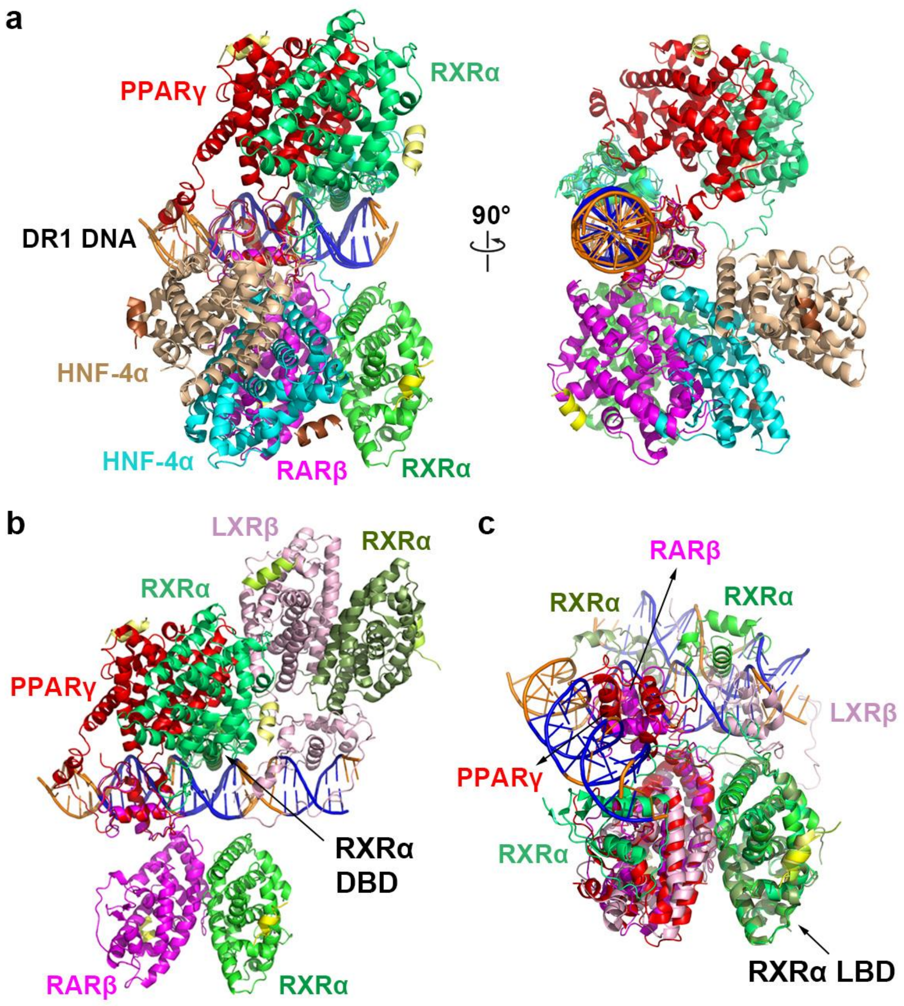

Supplementary Figure 5 Superimpositions of multi-domain NR crystal structures. (**a**) Superposed RARβ-RXRα heterodimer, PPARγ-RXRα heterodimer and HNF-4α homodimer structures by aligning the DR1 DNA sequences in two views. (**b** and **c**) Superposition of RARβ-RXRα, PPARγ-RXRα and LXRβ-RXRα structures, by aligning the common partner RXRα's DBD (**b**) or LBD (**c**), respectively.

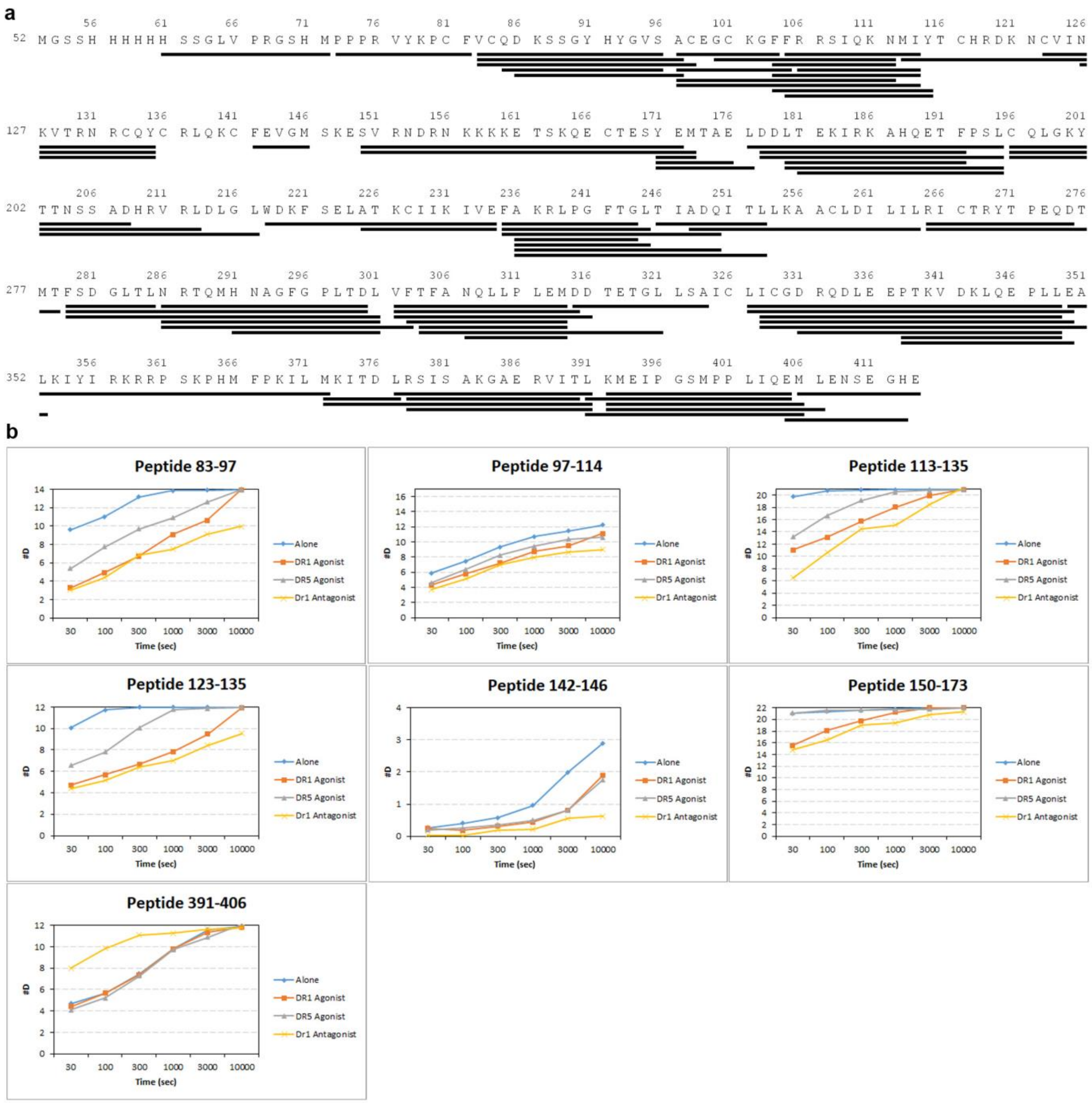

Supplementary Figure 6 Peptide coverage map for RARβ in the HDXMS studies (a) and individual uptake curves for selected regions (b).

Supplementary Table 1 Sequences of the primers used in this study.

RXRα cloning primers	Forward	AAAAAACATATGAACCCCGTCAGCAGC
	Reverse	AAAAAACTCGAGCTAAGTCATTTGGTGCGG
RARβ cloning primers	Forward	AAAAAAGCTAGCATGCCTCCCCCTCGAGTGTAC
	Reverse	AAAAAAGCGGCCGCTCATTCATGTCCTTCAGAATTCTC
RARβ E99A mutation primers	Forward	GGGGTCAGCGCCTGTGCTGGATGTAAGGGCTTTTTC
	Reverse	GAAAAAGCCCTTACATCCAGCACAGGCGCTGACCCC
RARβ R106A mutation primers	Forward	TGTAAGGGCTTTTTCGCTAGAAGTATTCAGAAG
	Reverse	CTTCTGAATACTTCTAGCGAAAAAGCCCTTACA
RARβ M113E mutation primers	Forward	AGAAGTATTCAGAAGAATGAAATTTACACTTGTCACCGA
	Reverse	TCGGTGACAAGTGTAAATTTCATTCTTCTGAATACTTCT
RARβ T116V mutation primers	Forward	CAGAAGAATATGATTTACGTCTGTCACCGAGATAAG
	Reverse	CTTATCTCGGTGACAGACGTAAATCATATTCTTCTG
RARβ K358E mutation primers	Forward	CTAAAAATTTATATCAGAGAAAGACGACCCAGCAAGCCT
	Reverse	AGGCTTGCTGGGTCGTCTTTCTCTGATATAAATTTTTAG
RARβ R359E mutation primers	Forward	AAAATTTATATCAGAAAAGAACGACCCAGCAAGCCTCAC
	Reverse	GTGAGGCTTGCTGGGTCGTTCTTTTCTGATATAAATTTT
RARβ R360E mutation primers	Forward	ATTTATATCAGAAAAAGAGAACCCAGCAAGCCTCACATG
	Reverse	CATGTGAGGCTTGCTGGGTTCTCTTTTTCTGATATAAAT
RARβ K358E/R359E/R360E mutation primers	Forward	CTAAAAATTTATATCAGAGAAGAAGAACCCAGCAAGCCTCACATG
	Reverse	CATGTGAGGCTTGCTGGGTTCTTCTTCTCTGATATAAATTTTTAG
RARβ S363E/K363E mutation primers	Forward	AGAAAAAGACGACCCGAAGAACCTCACATGTTTCCAAAG
	Reverse	CTTTGGAAACATGTGAGGTTCTTCGGGTCGTCTTTTTCT
